# Whole-Brain Mapping of Monosynaptic Afferent Inputs to Cortical CRH Neurons

**DOI:** 10.3389/fnins.2019.00565

**Published:** 2019-06-04

**Authors:** Shouhua Zhang, Fei Lv, Yuan Yuan, Chengyu Fan, Jiang Li, Wenzhi Sun, Ji Hu

**Affiliations:** ^1^Division of Physical Biology and Bioimaging Center, Shanghai Synchrotron Radiation Facility, Shanghai Institute of Applied Physics, Chinese Academy of Sciences, Shanghai, China; ^2^School of Life Sciences and Technology, ShanghaiTech University, Shanghai, China; ^3^University of Chinese Academy of Sciences, Beijing, China; ^4^iHuman Institute, ShanghaiTech University, Shanghai, China; ^5^Institute of Neuroscience, Chinese Academy of Sciences, Shanghai, China; ^6^Chinese Institute for Brain Research, Beijing, China

**Keywords:** corticotropin-releasing hormone, the anterior cingulate cortex, whole-brain mapping, monosynaptic inputs, rabies virus

## Abstract

Corticotropin-releasing hormone (CRH) is a critical neuropeptide modulating the mammalian stress response. It is involved in many functional activities within various brain regions, among which there is a subset of CRH neurons occupying a considerable proportion of the cortical GABAergic interneurons. Here, we utilized rabies virus-based monosynaptic retrograde tracing system to map the whole-brain afferent presynaptic partners of the CRH neurons in the anterior cingulate cortex (ACC). We find that the ACC CRH neurons integrate information from the cortex, thalamus, hippocampal formation, amygdala, and also several other midbrain and hindbrain nuclei. Furthermore, our results reveal that ACC CRH neurons receive direct inputs from two neuromodulatory systems, the basal forebrain cholinergic neurons and raphe serotoninergic neurons. These findings together expand our knowledge about the connectivity of the cortical GABAergic neurons and also provide a basis for further investigation of the circuit function of cortical CRH neurons.

## Introduction

Corticotropin-releasing hormone (CRH) is an important widely expressed neuropeptide with neuroendocrine and neurotransmitter properties, which is essential for brain function (Vale et al., 1981; Young, 2007). Since its initial identification and characterization, CRH has been indicated to play an important role in coordinating endocrine, autonomic, and behavioral responses to stress (Bale and Vale, 2004; Henckens et al., 2016). Previous studies have shown that a group of parvocellular neuroendocrine cells (PNCs) of the hypothalamic–pituitary–adrenal (HPA) axis the HPA axis, through somatic cells production and released into capillaries entering pituitary portal circulation, directly control pituitary corticotroph function, and downstream glucocorticoid secretion by the adrenal glands, of which most widely studied is CRH (Wamsteeker Cusulin et al., 2013). CRH has a major role in the regulation of the HPA axis, and it is the chief organizer of the body’s response to stress (Wang et al., 2011, 2013; Yang et al., 2015; Fang et al., 2016; Peng et al., 2017; Zhou and Fang, 2018). The anatomical distribution of CRH in the brain that this peptide is not only a key regulator of neuroendocrine stress, but also regulates neuronal activity in a neuromodulated manner (Dedic et al., 2018a). An increase of CRH expression is associated with several neurological disorders, such as Alzheimer’s disease (AD), major depression and anxiety disorders (Raadsheer et al., 1995). According to previous studies, CRH shows the most widespread expression in brain, but strongly expressed in several subcortical nuclei, such as paraventricular hypothalamic nucleus (PVN), amygdala, and the bed nucleus of the stria terminalis (BNST) (Chen et al., 2015; Dedic et al., 2018b; Deussing and Chen, 2018). Also, there is a subset of CRH neurons occupying a considerable proportion of the cortical GABAergic interneurons (Kubota et al., 2011). GABAergic interneurons are crucial in regulating the balance, flexibility, and functional architecture of cortical circuits (Markram et al., 2004; Klausberger and Somogyi, 2008). Their various intrinsic, synaptic, and dynamic properties allow interneurons to generate a rich range of inhibitory outputs (Jonas et al., 2004). Moreover, their different connectivity patterns confirm differential recruitment through appropriate inputs from specific brain regions (Somogyi et al., 1998; Buzsaki et al., 2004). They are of very importance in distinct forms of network oscillations that provide spatial-temporal frameworks to dynamically organize functional neural ensembles as well (Bartos et al., 2007; Klausberger and Somogyi, 2008; Taniguchi et al., 2011). The anterior cingulate cortex (ACC) is especially crucial for the performance of executive functions and emotional processing (Gasquoine, 2013; Kim et al., 2014; Meechan et al., 2015). There is evidence from electrophysiology and lesion studies indicating that the ACC plays an essential role in emotional self-control as well as focused problem-solving, error recognition, and adaptive response to changing conditions, which are central to intelligent behavior (Allman et al., 2001). It consists of several subdivisions, each with distinct functions that are provided by different input and output projections (Vogt and Paxinos, 2014). However, there is no comprehensive and systemic investigation of CRH neurons in the ACC. Therefore, characterizing the whole brain afferent pathways of CRH neurons in the ACC can expand the field of knowledge about molecular functions and neural circuit mechanism of CRH neurons.

The recent developed viral tracing system with modified rabies virus, which can map the monosynaptic afferents to a genetically defined neuronal subtype, has been applied to identify the whole-brain presynaptic partners of a specific type of neurons within a complex neural network (Wickersham et al., 2007; Ogawa et al., 2014; Grealish et al., 2015; Hu et al., 2016). Here, we applied such viral tracing system to illustrate the whole-brain afferent inputs of the ACC CRH neurons and investigated what kind of information it integrated from several important upstream brain regions. We identified the presynaptic partners of ACC CRH neurons from neocortex and thalamus. Also, we found that hippocampal information, amygdala and olfactory areas sent direct projections to the ACC CRH neurons. Interestingly, two neuromodulatory systems, the basal forebrain cholinergic system and raphe serotoninergic system, provide direct innervation onto the ACC CRH neurons. Therefore, our results should be valuable to guide further investigations of the functional roles of the ACC CRH neurons, such as the normal and neurological disease states.

## Materials and Methods

### Ethical Approval

This study was carried out in accordance with the recommendations of the guidelines issued by the Institutional Animal Care and Use Committees (IACUC) at Wuhan Institute of Physics and Mathematics, the Chinese Academy of Sciences, China. The protocol was approved by IACUC at ShanghaiTech University. Every effort was made to ensure the mice used were treated humanely and any discomfort was kept to a minimum.

### Animals

All mice were housed under a 12/12 day/night cycle at the temperature of 22–25°C, with *ad libitum* access to rodent food and water freely available in environmentally controlled conditions. The mice used in the study were adult (8–15 weeks) CRH-ires-Cre knock-in mice (Stock No. 012704) (Jackson Laboratory, Bar Harbor, ME) and C57BL/6 mice (N/A) (Shanghai Model Organisms).

### Viral Microinjection and Stereotactic Surgery

All the viruses used in the *trans-*synaptic retrograde tracing experiments included AAV-CAG-DIO-TVA-GFP (AAV2/9, 1.7 × 10^13^ genomic copies per ml), AAV-CAG-DIO-RG (AAV2/9, 6.8 × 10^12^ genomic copies per ml), and EnvA-pseudotyped, glycoprotein (RG)-deleted and DsRed-expressing rabies virus (RV-EvnA-DsRed, RV) (5.0 × 10^8^ genomic copies per ml), which were packaged and provided by F. Xu (Wuhan, China). Surgical procedures generally followed previous studies (Liu et al., 2014; Hu et al., 2017). In brief, mice were anesthetized under isoflurane, kept warm (37°C) with an electric heating pad (BrainKing Biotech, Beijing), and placed in a stereotaxic apparatus to adjust the skulls of experimental mice in parallel to the reference panel. Using a microsyringe pump (Nanoject III #3-000-207, DRUMMOND), 150 nl∼300 nl of AAV-CAG-DIO-TVA-GFP and AAV-CAG-DIO-RG were stereotaxically injected (20 nl/min) into the bilateral ACC (+1.10 mm AP, ± 0.20 mm ML, −1.30 mm DV, relative to Bregma) of CRH-ires-Cre mice and C57BL/6 mice, an additional 5 min being allowed for viral particles to diffuse away from the injection site before the pump was slowly withdrawn. After 2 weeks of helper viruses expression, 300 nl of RV-EvnA-DsRed was injected into the same location of the previous injection of CRH-ires-Cre mice. C57BL/6 mice were directly perfused.

### Histology and Image Analysis

One week after injection of the rabies virus, CRH-ires-Cre mice were deeply anesthetized by intraperitoneal injection of an overdose of pentobarbital and then intracardially perfused with 0.9% saline solution followed by 4% paraformaldehyde (PFA) in PBS. After 2 h of post-fixation in 4% PFA, brain samples were transferred to 30% sucrose (m/v) in 1 × PBS over one night. Then brains prepared with the optimum cutting temperature compound (O.C.T Compound) were sectioned coronally in 50 μm thickness on a freezing microtome (Leica CM1900). One out of every three sections was counterstained with nucleus dye DAPI (Molecular Probes, Eugene, OR, United States) and these sections were imaged for all subsequent analyses with an Olympus VS120 microscope. For the quantifications of starter cells and afferent input cells, we divided the boundaries of the subregions, according to the Allen Institute’s reference atlas (Lein et al., 2007). Further data analyses were carried out using Olympus analysis software, ImageJ software and GraphPad Prism7. All values were presented as the Mean ± SEM. To characterize the rabies-labeled cells in different regions, some of the remaining sections were selected for immunostained with various antibodies, including the primary goat anti-choline acetyltransferase (ChAT) antibody (1:200, Abcam, United Kingdom), primary mouse anti-tyrosine hydroxylase (TH) antibody (1:1000, Abcam, United Kingdom), primary rabbit anti-tryptophan hydroxylase 2 (Tph2) antibody (1:1000, Abcam, United Kingdom), Alexa Fluor 488 donkey anti-rabbit second antibody (1:1000, Abcam, United Kingdom), Alexa Fluor 488 goat anti-mouse second antibody (1:1000, Abcam, United Kingdom) and Alexa Fluor 488 donkey anti-goat second antibody (1:1000, Abcam, United Kingdom). Briefly, the sections were first blocked with 3% BSA in PBS-0.3% Triton X-100 for 30 min and incubated with the primary antibodies for 48 h at 4°C. After washing, the sections were incubated with second antibodies for 2 h at room temperature. Brain sections were imaged with 20× and 60× objectives on a confocal microscope (Nikon Ti-E+A1 R SI).

## Results

### Strategies for Tracing Monosynaptic Inputs to the ACC CRH Neurons

**FIGURE 1:**
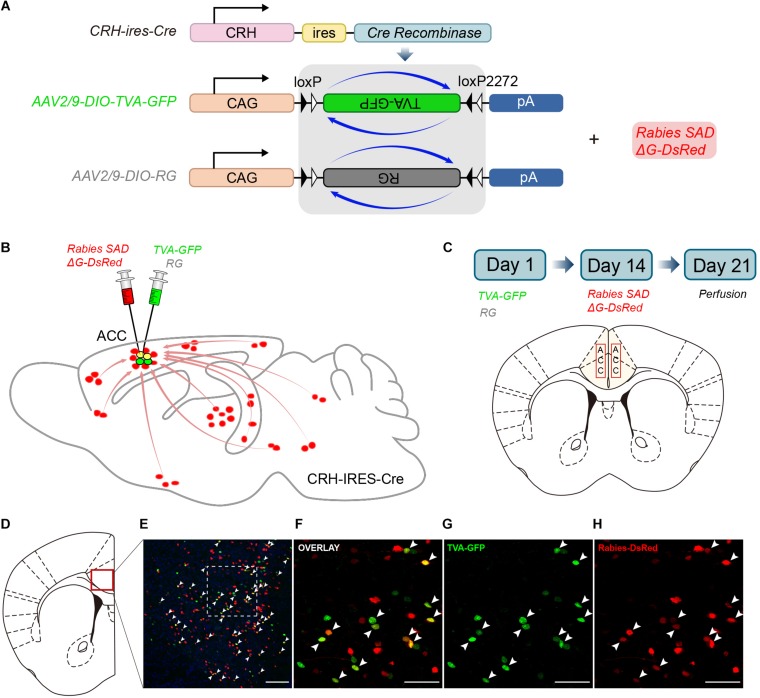
Monosynaptic inputs to the ACC CRH neurons using the rabies virus and CRH-ires-Cre knock-in mice. **(A)** The AAV helper virus and EnvA pseudotyped glycoprotein (G)-deleted rabies virus. **(B)** Combination of the two virus system and CRH-ires-Cre mice allows for brain-wide labeling of monosynaptic inputs (red) to CRH neurons in the ACC. **(C)** Timeline of virus injection for retrograde *trans-*synaptic tracing and the schematic of the anatomical localization of the ACC. **(D–H)** Representative confocal images of the injection site. As the starter cells express both GFP and DsRed fluorescent proteins, they are shown in yellow in the image. Panel **(D)** shows the diagram of the ACC section. Red box indicates the region shown in **(E)**. Panels **(F–H)** show enlarged views of the white-boxed region in **(E)**. Scale bars: 100 μm in **(E)** and 50 μm in **(F–H)**.

CRH-ires-Cre mice, a genetically engineered mouse line, were used to target CRH neurons specifically. We utilized rabies-based viral strategy to map the whole-brain monosynaptic inputs to the ACC CRH neurons (Wall et al., 2010; Figure 1A). The rabies virus was pseudotyped with an avian virus envelope protein (EnvA), so they could not infect mammalian cells without a cognate receptor (e.g., TVA) (Watabe-Uchida et al., 2012). In addition, RG gene, which was required for transsynaptic spread, had been genetically replaced by fluorescent protein DsRed.

In Day 1, the mixture of two helper viruses (equal amount of AAV2/9-DIO-TVA-GFP and AAV2/9-DIO-RG) was stereotaxically micro-injected into the bilateral ACC of CRH-ires-Cre mice (Figures 1B,C). The helper viruses were Cre dependent, so TVA-GFP and RG proteins were only expressed in CRH neurons where the Cre recombinase existed. In order to verify the specificity of the helper virus, we also injected the same amount of virus into the ACC of C57BL/6 mice. After 2 weeks, C57BL/6 mice were perfused and the brains were sectioned at 50 μm. As for CRH-ires-Cre mice, the rabies virus, Rabies SADΔG-DsRed was injected into the same area. Rabies virus only infected CRH cells expressing the TVA receptor and then retrograde spread to the upstream cells with the help of RG. The mice were sacrificed seven days after the last injection; then the whole brains were sectioned at 50 μm for further anatomic analysis. Starter cells were both GFP^+^ (from the TVA-GFP fusion) and DsRed^+^ (from rabies virus) (Supplementary Figure S1), whereas their presynaptic partners were only DsRed^+^ (Figures 1D–H). 2010 ± 200 (Mean ± SEM) GFP^+^ cells were counted in CRH-ires-Cre mice, whereas no GFP^+^ cell in C57BL/6 mice (Supplementary Figure S2). In summary, the joint use of CRH-ires-Cre line and rabies virus validated our strategy of monosynaptic retrograde tracing of the ACC CRH neurons.

### Overview of the Whole-Brain Inputs to the ACC CRH Neurons

**FIGURE 2:**
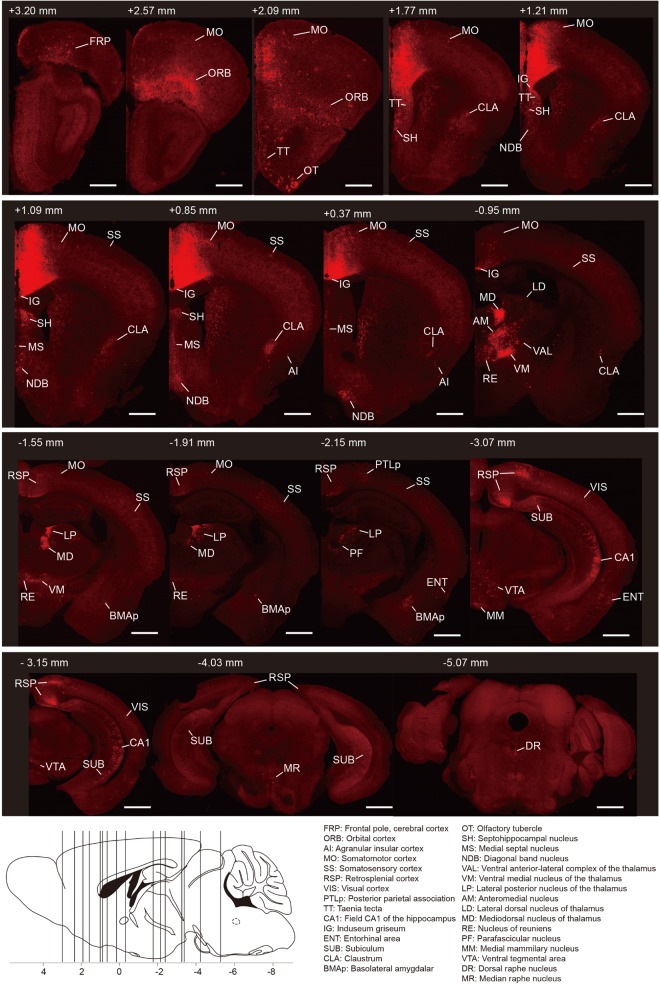
Whole-brain distributions of monosynaptic inputs to the ACC CRH neurons. Representative coronal sections showing labeling of monosynaptic inputs to the ACC CRH neurons. For some sections, only the unilateral side is shown. Scale bar, 1 mm. Bottom-left: illustration of the anatomical localization of the sections shown above.

To generate the overall distribution of the rabies-labeled presynaptic partners of the ACC CRH neurons, we imaged serial whole-brain coronal sections (Figure 2). Then we identified each input area manually based on the Allen Institute’s reference atlas and found the ACC CRH neurons integrate monosynaptic inputs from widespread brain regions, ranging from the cerebral cortex to the hindbrain (Bregma +3.2 mm∼ −5.4 mm).

**FIGURE 3:**
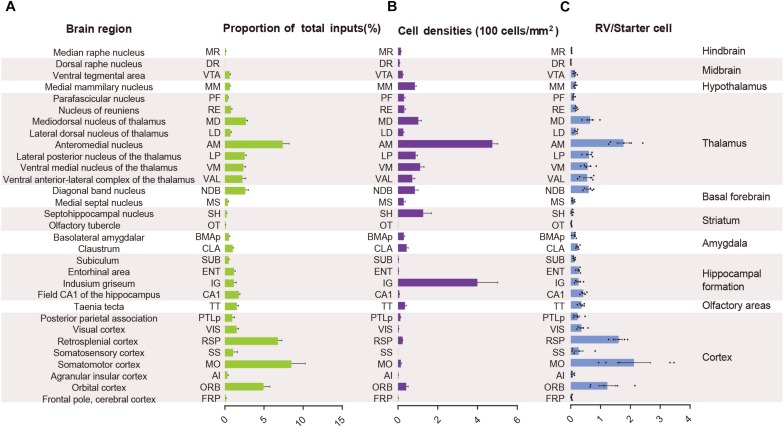
Statistical analysis of the monosynaptic inputs to the ACC CRH neurons. **(A)** The proportion of input neurons in each brain region. The values are normalized by the total number of input neurons. **(B)** Cell density of input neurons in each brain region. **(C)** Ratio of rabies-labeled neurons in each brain region to starter neurons in the ACC. Mean ± SEM (*n* = 5 CRH-ires-Cre mice).

In order to quantify each upstream brain area, all the inputs were divided into 31 regions of interest belonging to 11 large brain regions. Then we counted the number of input neurons in each area and computed their proportions of total inputs (Figure 3A). The results showed that most of the afferents to the ACC CRH neurons originated from the cortex. Among different cortical subregions, the majority of the projections (83.75% ± 1.56%, Mean ± SEM) were received from somatomotor (MO), retrosplenial (RSP) and orbital cortex (ORB) (Figures 2, 3A) and the ACC afferents in the secondary motor cortex (MOs) were more than that in the primary motor cortex (Mop) (Supplementary Figure S4A). The thalamus was the second largest inputs source (34.42% ± 2.30%, Mean ± SEM), in which over a third of inputs (38.45% ± 1.90%, Mean ± SEM) come from the anteromedial nucleus (AM) (Figures 2, 3A). Additionally, hippocampal formation and amygdala accounted for minor direct projections (10.97% ± 1.44%, Mean ± SEM) to the ACC CRH neurons (Figures 2, 3A). There were also a few other areas in midbrain and hindbrain that yielded weak innervations (1.39% ± 0.24%, Mean ± SEM) (Figures 2, 3A). We further calculated the cell densities of input neurons in each area (Figure 3B) and the ratios of rabies-labeled neurons to starter neurons in the ACC (Figure 3C). For some brain regions with high cell density, such as the ORB, RSP, AM, and the mediodorsal nucleus of the thalamus (MD), we showed their high-resolution pictures in the Supplementary Figure S3.

### The ACC CRH Neurons Receive Extensive Cortical Inputs

We found significant monosynaptic inputs from cortical areas (Figures 2, 3A). In the neocortex, rabies-labeled neurons were widely distributed across cortical areas, including the frontal pole (FRP), ORB, agranular insular cortex (AI), MO, somatosensory cortex (SS), RSP, visual cortex (VIS), and posterior parietal association (PTLp) (Figures 2, 6).

Overall, the MO, RSP, and ORB comprised a significant portion of the cortical projection to the ACC which were about 8.50% ± 1.79%, 6.79% ± 0.51%, and 4.94% ± 0.81% (Mean ± SEM), respectively (Figure 3A). Among them, the RSP was implicated in a wide range of cognitive functions including navigation, episodic memory, and imagining future events in human fMRI studies (Spreng et al., 2009; Vann et al., 2009). Furthermore, the VIS, SS, and PTLp occupied a medium proportion that were about 1.51% ± 0.26%, 1.06% ± 0.57%, and 0.96% ± 0.30% (Mean ± SEM) (Figure 3A). We also found that input neurons in the FRP and AI accounted for only about 0.31% ± 0.12% and 0.13% ± 0.04% (Mean ± SEM) of the rabies-labeled neurons (Figure 3A). Notably, the major input neurons in cortical areas were found in deep layer 2/3 or layer 5, such as in the MO, SS, PTLp, and VIS (Supplementary Figure S4). These results suggested that the ACC CRH neurons received projections primary from the motor cortex and sensory cortex, which may provide insights for the further investigation of the ACC CRH neurons in somatic movement and cognition processing.

### The ACC CRH Neurons Receive Strong Thalamic Inputs

The thalamus has complex functions, generally viewed as a relay station to transfer and integrate sensory signals, including motor signals to the cortical areas, and the modulation of sleep, alertness, learning, and decision-making (Mitchell, 2015; Feng et al., 2017; Hwang et al., 2017). The thalamus is globally connected with different cortical areas, yet the cell types of connections between each thalamus nucleus and distributed cortical regions remain elusive.

Our results demonstrated that the thalamus was the second largest inputs source (34.42% ± 2.30%, Mean ± SEM) of the ACC CRH neurons, showing pervasive and widespread monosynaptic input neurons (Figures 2, 3). Among different sub-nuclei of the thalamus, more than a third of projections (38.45% ± 1.90%, Mean ± SEM) were received from the AM (Figure 3A). The anterior thalamic nuclei have been shown to support multiple and complementary forms of learning and social defeat-associated contextual fear memory (Rangel et al., 2018). Our results may provide the new perspectives for the functional role of the ACC CRH neurons in learning and memory process. Also, the mediodorsal nucleus (MD), lateral posterior nucleus (LP), ventral medial nucleus (VM) and ventral anterior-lateral complex (VAL) of thalamus contributed almost equally and made moderate direct projections to the ACC CRH neurons (Figures 2, 3A). Furthermore, many regions of the thalamus were sparsely labeled, including the parafascicular nucleus (PF), the nucleus of reuniens (RE) and the lateral dorsal nucleus of the thalamus (LD) (Figure 2). Our results revealed that thalamus sent broad projections to CRH neurons in the ACC, indicating that the cortical CRH neurons should be considered in the future exploration of the thalamic functions in the processing of learning, memory, and cognition.

### The ACC CRH Neurons Receive Inputs From the Basal Forebrain

**FIGURE 4:**
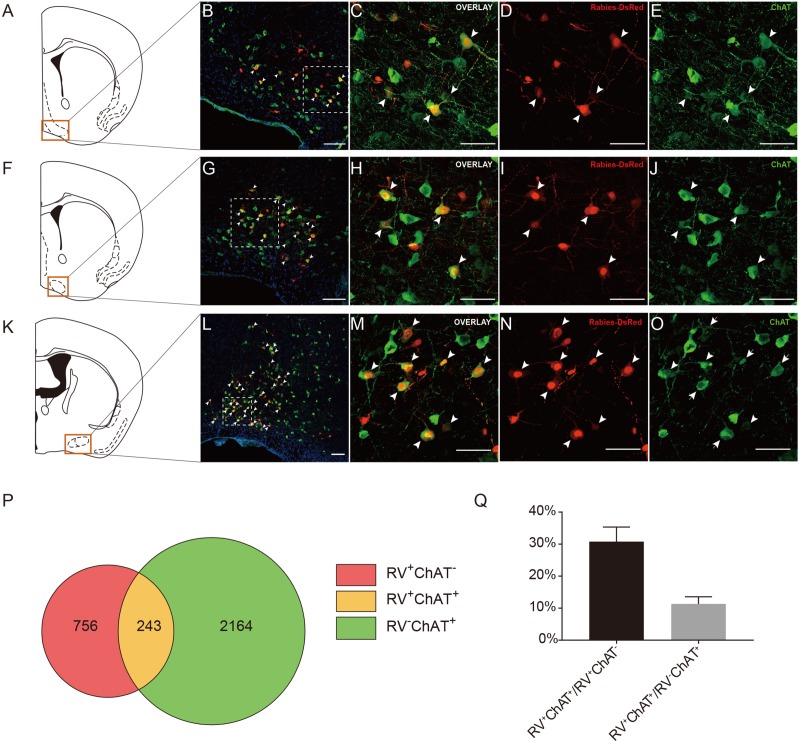
Immunochemical characterization and quantification of rabies-labeled neurons in NDB presynaptic to the ACC CRH neurons. **(A–O)** Immunostaining of choline acetyltransferase (ChAT) in brain slices with rabies-labeled diagonal band nucleus (NDB) neurons. Panels **(A,F,K)** show three diagrams of anterior-posterior NDB sections. Red boxes indicate the regions shown in **(B,G,L)**. Panels **(C–E)** show enlarged views of the white-boxed regions in **(B)**. Panels **(H–J)** show enlarged views of the white-boxed regions in **(G)**. Panels **(M–O)** show enlarged views of the white-boxed regions in **(L)**. The rabies-labeled cells and ChAT^+^ cells are shown in red and green, respectively. Scale bars: 100 μm in **(B,G,L)** and 50 μm in **(C,D,E,H,I,J,M–O)**. **(P,Q)** Pie chart **(P)** and bar chart **(Q)** analyses illustrating the proportions of RV^+^ChAT^+^ neurons in RV^+^ neurons or ChAT^+^ neurons. Mean ± SEM (*n* = 5 CRH-ires-Cre mice).

Previous studies have demonstrated that the basal forebrain is a complex nucleus which provides GABAergic, glutamatergic neurons and cholinergic inputs to cortical areas (Gritti et al., 1997; Hur and Zaborszky, 2005; Zaborszky et al., 2015). Functionally, it has suggested that the projections from the basal forebrain to the cortex played an important role for cortical states and was also implicated in attention, sensory processing and learning (Buzsaki et al., 1988; Everitt and Robbins, 1997; Duque et al., 2000; Fuller et al., 2011; Pinto et al., 2013). Consistent with these studies, our retrograde tracing results indicated that the basal forebrain provided significant inputs to the ACC CRH neurons (Figures 2, 3). Moreover, the cholinergic neurons have been associated with plasticity and selective attention (Wenk, 1997; Rasmusson, 2000). The dysfunction of the cholinergic neurons in the basal forebrain has also been related to neurological disorders such as Alzheimer’s disease (AD) and schizophrenia (Cuello et al., 2010; Marra et al., 2012; Burke et al., 2013). To further understand the nature of the basal forebrain projections to the ACC, we performed neurochemical characterization of rabies-labeled input neurons to explore the potential difference of cell types in diagonal band nucleus (NDB) as it comprised a major portion of the basal forebrain inputs to the ACC (Figure 3A). Immunochemical staining against ChAT allowed for the identification of cholinergic NDB-projecting cells (Figures 4A–O). Interestingly, we found that 30.79% ± 4.08% (Mean ± SEM) of the rabies-labeled NDB cells were cholinergic (Figures 4P,Q), which suggested that the ACC CRH neurons may contribute to some vital brain functions of the cholinergic circuit. The basal forebrain contains cholinergic, GABAergic, glutamatergic and peptidergic neurons (Amaral and Kurz, 1985; Freund and Antal, 1988; Gulyás et al., 1990). Maybe the ChAT negative neurons labeled by rabies virus were other cell types.

### The ACC CRH Neurons Receive Inputs From the Midbrain and Hindbrain

There were a few areas in the midbrain and hindbrain that provided weak but important innervations. The dopamine (DA) neurons originated from either the ventral tegmental area (VTA) or substantia nigra pars compacta (SNc) have been proposed to have complex and multifaceted functions, including modulating appetitive, reward-related behaviors (Holly and Miczek, 2016; Zhang et al., 2017). There are many brain areas conveying information to DA neurons, and DA neurons, in turn, send projections to the prefrontal cortex, thalamus, hippocampus, amygdala and striatum, demonstrating the “feedback” nature of this circuit (Avery and Krichmar, 2017). However, many questions remain to date regarding cortical inputs to the dopaminergic system.

**FIGURE 5:**
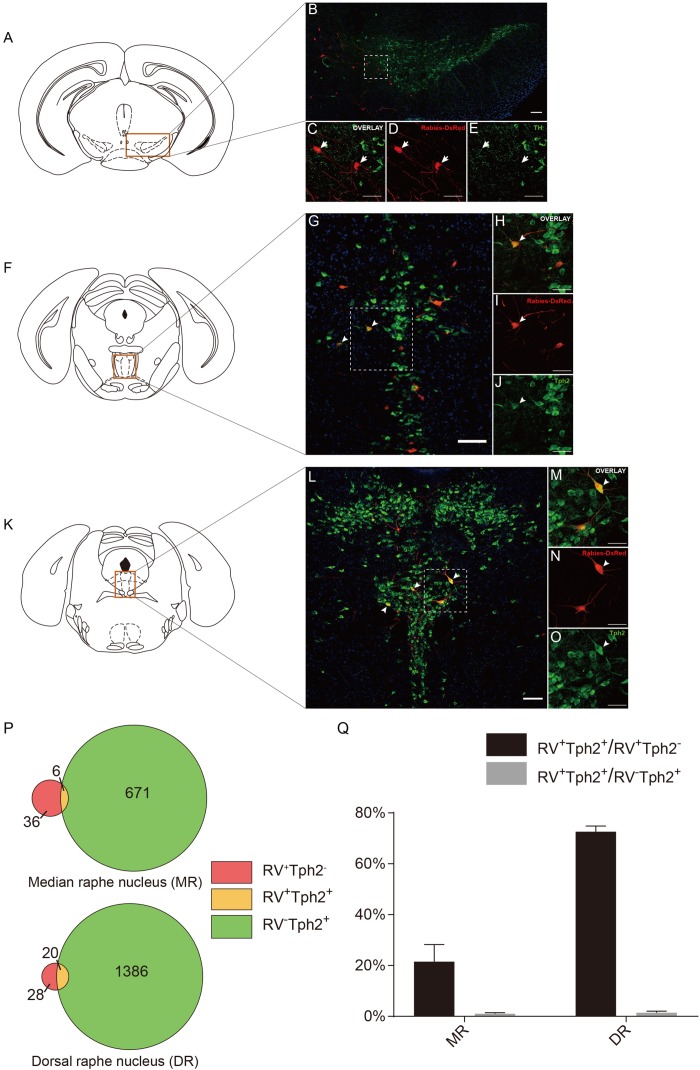
Neurochemical characterization of rabies-labeled, ACC-projecting neurons in midbrain and hindbrain. **(A–E)** Immunostaining of tyrosine hydroxylase (TH) in brain slices with rabies-labeled ventral tegmental area (VTA) neurons. Panel **(A)** shows the diagram of VTA section. Red box indicates the region shown in **(B)**. Panels **(C–E)** show enlarged views of the white-boxed regions in **(B)**. The rabies-labeled cells and TH^+^ cells are shown in red and green, respectively. None of the rabies-labeled cells are TH positive (arrow). **(F–J)** Immunostaining of tryptophan hydroxylase 2 (Tph2) in brain slices with rabies-labeled median raphe nucleus (MR) neurons. Red box indicates the region shown in **(G)**. Panels **(H–J)** show an enlarged view of the white-boxed regions in **(G)**. The rabies virus-labeled cells and Tph2^+^ cells are shown in red and green, respectively. A fraction of rabies-labeled cells are Tph2 positive (arrowheads). **(K–O)** Immunostaining of Tph2 in brain slices with rabies-labeled dorsal raphe nucleus (DR) neurons. Panel **(K)** shows the diagram of DR section. Red box indicates the region shown in **(L)**. Panels **(M–O)** show enlarged views of the white-boxed regions in **(L)**. The rabies virus-labeled cells and Tph2^+^ cells are shown in red and green, respectively. A significant part of rabies-labeled cells are Tph2 positive (arrowheads). Scale bars: 100 μm in **(B,G,L)** and 50 μm in **(C,D,E,H,I,J,M–O)**. **(P,Q)** Pie chart **(P)** and bar chart **(Q)** analyses illustrating the proportions of RV^+^Tph2^+^ neurons in RV^+^ neurons or Tph2^+^ neurons in MR and DR. Mean ± SEM (*n* = 5 CRH-ires-Cre mice).

To further provide the new perspectives for the function of the dopaminergic system, we examined the cell type of rabies-labeled neurons in the VTA by immunostaining experiments (Figures 5A–F). The TH, a marker of DA neurons was used to identify DA neurons in the VTA. After immunostaining, the densest TH positive staining was observed in the middle and anterior portion of the VTA, including parabrachial pigmented area (PBP) and parafasciculus retroflexus area (PFR) (Figure 5B). We observed that none of the rabies-labeled cells are TH positive (Figures 5C–E), which may imply not the CRH neurons in the ACC, but other types, involve in the VTA dopaminergic circuitry. Although DA neurons in the VTA are widely studied, GABAergic and glutamatergic neurons are also abundant (Faget et al., 2016). So in this *trans-*synaptic retrograde tracing experiment, the TH negative neurons maybe GABAergic or glutamatergic.

**FIGURE 6:**
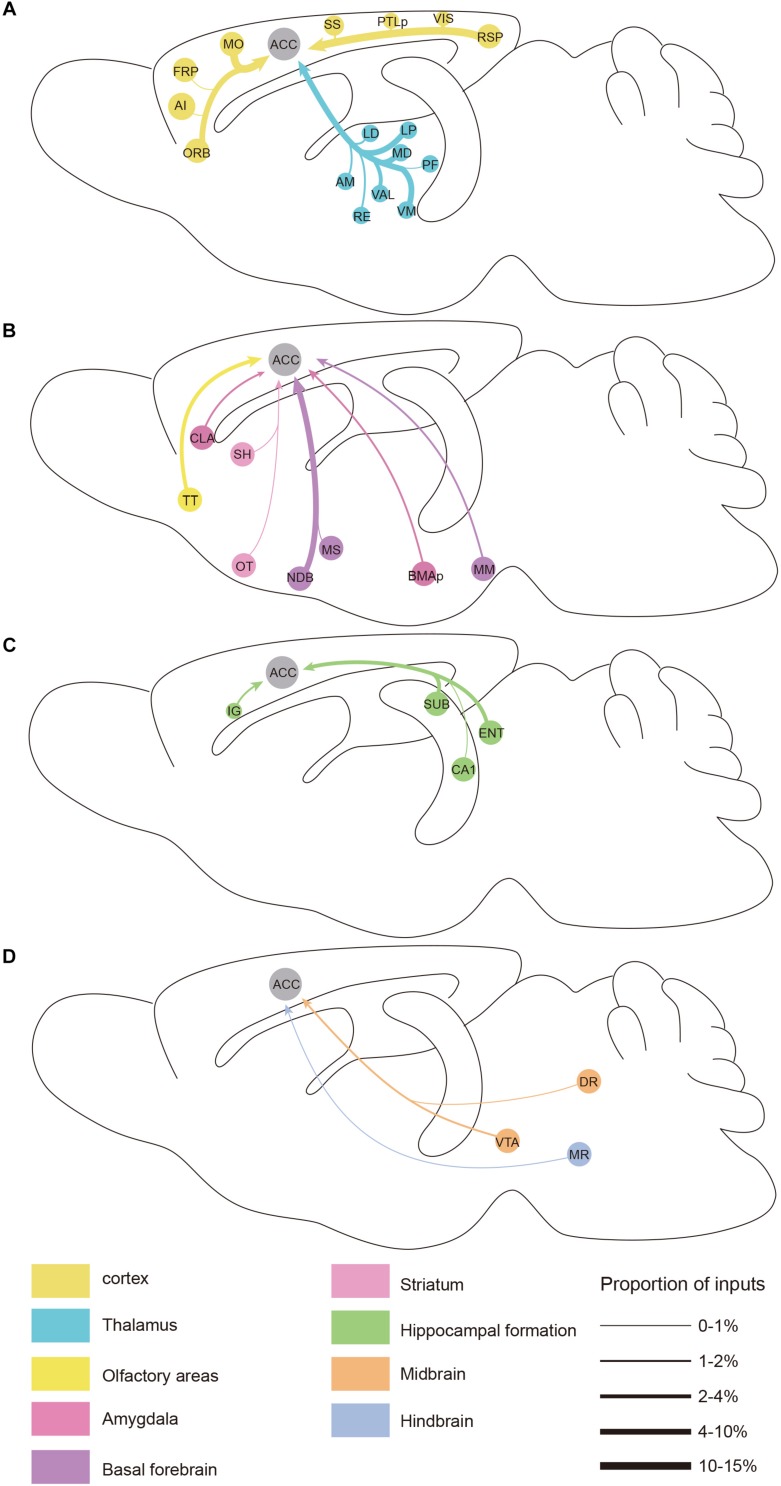
Summarized whole brain monosynaptic inputs to the ACC CRH neurons. **(A)** Schematic of the cortical and thalamic inputs. **(B)** Schematic of the olfactory areas and forebrain subcortical inputs. **(C)** Schematic of the hippocampal inputs. **(D)** Schematic of inputs from the midbrain and hindbrain. Brain regions of the same color belong to the same brain structure shown below. The thickness of each line indicates the proportion of input neurons in each area as defined at the bottom right.

Serotonin (5-hydroxytryptamine, 5-HT), another neuromodulator, has been proposed to have an essential impact on brain functions. Several studies have revealed 5-HT’s involvement in predicting punishment or harm aversion, impulsivity, stress and anxiety, and a wide variety of functions including emotion, sleep, reward, attention, and memory (Millan, 2003; Winstanley et al., 2003; Cools et al., 2008; Crockett et al., 2008, 2015; Jasinska et al., 2012; Nakamura, 2013; Seyedabadi et al., 2014; Quentin et al., 2018). The raphe nuclei, including the median raphe (MR) and dorsal raphe (DR), produce the major serotonergic populations in the central nervous system (CNS). Previous investigations have proposed that the principal targets of the raphe nucleus are “limbic cortices” including the ACC. Also, there was evidence demonstrating that the raphe nucleus received input back from “limbic cortices” (Vertes and Linley, 2008; Pollak Dorocic et al., 2014). Interestingly, the MR and DR serotonergic projections are two distinct systems differing in their morphology and physiology (Hensler, 2006). Previous studies revealed that there are few or partially overlapping in the final projections in the cortex of these two sets of serotonergic nuclei (Hensler, 2006; Vertes and Linley, 2008).

To further identify the disparity between the monosynaptic inputs to the ACC CRH neurons in the MR and DR, we carried out the immunostaining experiments (Figures 5F–O) and made some statistical analysis (Figures 5P,Q) afterward. Tph2 immunostaining was performed to identify 5-HT neurons in the raphe nucleus. Our results revealed, among rabies-labeled neurons, the proportion of 5-HT neurons in the MR (21.49% ± 6.05%, Mean ± SEM) was significantly lower than the DR (72.61% ± 2.24%, Mean ± SEM) (Figure 5Q). Besides 5-HT neurons, GABAergic neurons in the DR also project to the forebrain (Bang and Commons, 2012). These RV^+^TPH2^–^ cells we labeled may be GABAergic neurons. This difference may be quite important for understanding how these two distinct serotonergic systems modulate the limbic system in normal brain and psychiatric disorders. This may give us a more rigorous cue of the circuitry of 5-HT neurons in the brain and help to understand how serotonergic and CRH system interconnected in normal and disease conditions.

## Discussion

The ACC has been linked to some of the most pivotal behaviors, such as decision-making, conflict monitoring and pain processing (Kolling et al., 2016; Xiao and Zhang, 2018). In order to understand the circuit mechanism associated with these behaviors more accurately, it is necessary to investigate the whole-brain inputs to the specific cell-type neurons in the ACC. In the present study, our viral tracing results efficiently mapped a comprehensive list of monosynaptic inputs to the ACC CRH neurons. We demonstrated the ACC CRH neurons receive major direct inputs from cortical regions and thalamus nucleus. Furthermore, we showed that the cholinergic system and serotoninergic system in the basal forebrain and raphe nuclei, respectively, provide neuromodulatory inputs to the ACC CRH neurons. Though our results were almost identical to those afferents of ACC neurons labeled by traditional reverse tracer FG, in a few brain areas labeled by FG, there were no RV^+^ cells in our results, such as the substantia nigra, *pars compacta* (SNc) (Fillinger et al., 2017).

### Monosynaptic Reverse Tracing by Rabies Virus With Cre Recombinase Transgenic Mice

Compared with conventional retrograde tracing techniques, rabies virus system allows for mapping of monosynaptic inputs to defined neuronal subtypes by combining Cre-loxp system. In this study, we used CRH-ires-Cre mice to target CRH neurons specifically. Through the joint use of CRH-ires-Cre line and rabies virus system, we exclusively distinguished the CRH cell-specific inputs from the general inputs to ACC. As previous studies showed, this method with the combination of these two systems was efficient in labeling the monosynaptic inputs of the cell-type specific neurons (Watabe-Uchida et al., 2012; Pollak Dorocic et al., 2014; Weissbourd et al., 2014). During the data analysis, we found that some patterns of labeling produced by the rabies virus are not in accordance with known brain connectivity, but the rabies-labeled brain areas projecting to ACC CRH neurons in all mice in our experiments were consistent, for example, the labeling of the olfactory tubercle. In addition, the monosynaptic tracing of CRH neurons revealed a comprehensive atlas of the presynaptic partners of the ACC CRH neurons with a high resolution. We mapped average 12,214 neurons per animal and showed the ACC CRH neurons receive direct inputs from raphe nuclei even though there were just a minimal number of rabies-labeled inputs (0.15% roughly). Moreover, the number of starter cells is available, which makes it possible to carry out the statistical analysis and generate a quantitative and precise map of whole-brain monosynaptic inputs to the ACC CRH neurons. On the other hand, there are still a few drawbacks that may influence the statistical results because of the limitation of rabies retrograde tracing. For example, some CRH neurons expressing TVA-GFP but not express RG due to two separate helper virus. Consequently, the number of starter cells may be overestimated, then the ratios of rabies-labeled neurons in each brain region to starter neurons may be underestimated. Apart from that, there are a few TVA-GFP positive but RV negative neurons in the ACC, suggesting that this system has certain limitations on the transduction efficiency of the rabies virus. The characteristics of RV-based monosynaptic retrograde tracing strategy make it more suitable for exploring the long-range brain neuronal connectivity by mapping the monosynaptic inputs onto defined cell types in a specific region, facilitating related more in-depth research in the future.

### Implications for the Role of the ACC CRH Neurons in Pain Processing

Pain is a distressing sensory and emotional experience often associated with intense or damaging stimuli alert the individual to withdraw from harmful damage (Bushnell et al., 2013; Bliss et al., 2016). Anatomical and physiological studies have revealed that the ACC and other cortical areas, including the somatosensory cortex, prefrontal cortex and the insular cortex, are activated by various painful stimuli (Talbot et al., 1991; Schnitzler and Ploner, 2000; Xiao and Zhang, 2018). Previous studies showed that CRH had an important function on the modulation of pain resulted from bone cancer or inflammatory nociceptive stimuli and acted on important brain structures in pain regulatory (Lariviere and Melzack, 2000; Lariviere et al., 2011; Fan et al., 2015). Under pain processing, neurons in the thalamus play important effects in relaying the ascending information to the ACC, somatosensory cortex, prefrontal cortex, insular cortex and amygdala (Apkarian et al., 2005; Zhuo, 2014; Bliss et al., 2016). Then, the ACC, as a critical brain area involved in pain processing, projecting to periaqueductal gray, prefrontal cortex, and insular cortex. As well, neurons in the deep layers in the ACC innervate directly or indirectly to the spinal dorsal horn. All of these connections make up a spinal dorsal horn–thalamus–cortex–spinal dorsal horn loop in pain processing (Fuchs et al., 2014; Bliss et al., 2016). Our results showed that CRH neurons in the ACC receive a great number of projections from the somatosensory cortex and thalamus. Besides, amygdala, which has been linked to emotion, also provides moderate inputs to CRH neurons in the ACC. These connections may point out the ACC CRH neurons take part in the signal transmission and emotion storage in pain processing.

### Implications for the Role of the ACC CRH Neurons in Emotion

The function of emotion has been described as to decouple stimulus and response, thus modulating cognition to allow for a suitable adaptation to the environment (Scherer, 1994; Brosch et al., 2013). Emotion has a critical contribution to perceiving the world, the enhancement of memory and decision-making (Brosch et al., 2013; Desmedt et al., 2015). Because of its complexity, there are a set of neural mechanisms that modulate many brain regions simultaneously in emotional behavior and neuromodulatory systems play a crucial part in the experience and expression of emotion (Fellous, 1999; LeDoux, 2000). Neuromodulatory systems, including the cholinergic system, serotonergic system, noradrenergic and dopaminergic system, are suggested to be important for many crucial behaviors, such as rewards, aversion, risks, cooperation, and novelty (Krichmar, 2008). The cholinergic system has been linked with various functions including attention, learning and memory, sleep, cognition, and emotion (Hasselmo, 2006; Platt and Riedel, 2011; Picciotto et al., 2012; Ballinger et al., 2016; Mu and Huang, 2019). Besides, several studies have revealed 5-HT’s involved in many brain functions, such as emotion, reward, attention, and memory (Cools et al., 2008; Nakamura, 2013; Seyedabadi et al., 2014; Li et al., 2016; Quentin et al., 2018). The interactions between these systems and the other regions, like the ACC, frontal cortex, hippocampus, sensory and striatum, provide a foundation for higher cognitive functions, including emotion (Avery and Krichmar, 2017). Also, previous studies proposed that the ventral hippocampus (anterior in primates) relates to emotion (Fanselow and Dong, 2010; Grigoryan and Segal, 2016). Our retrograde tracing revealed that the ACC CRH neurons receive projections from (1) the cholinergic neurons in the basal forebrain (about 31% rabies-labeled neurons in the NDB); (2) the serotonergic neurons in the raphe nucleus (about 73% rabies-labeled neurons in the DR and 21% in the MR); (3) neurons in the ventral hippocampus (mainly in the CA1). These results give us more indications about the functions of the ACC CRH neurons with neuromodulatory systems and hippocampus in emotion processing, illustrating such connection is a critical component for developing a circuit-level understanding of emotion, even other higher cognitive functions.

## Data Availability

The raw data supporting the conclusions of this manuscript will be made available by the authors, without undue reservation, to any qualified researcher.

## Ethics Statement

This study was carried out in accordance with the recommendations of the guidelines issued by the Institutional Animal Care and Use Committees (IACUC) at Wuhan Institute of Physics and Mathematics, the Chinese Academy of Sciences, China. The protocol was approved by IACUC at ShanghaiTech University. Every effort was made to ensure the mice used were treated humanely and any discomfort was kept to a minimum.

## Author Contributions

JH, JL, WS, YY, and SZ conceptualized the project. SZ and FL performed the majority of experiments. JH, SZ, FL, and CF analyzed the data. JH, SZ, and FL wrote the manuscript with the participation of all other authors.

## Conflict of Interest Statement

The authors declare that the research was conducted in the absence of any commercial or financial relationships that could be construed as a potential conflict of interest.

## Supplementary Material

FIGURE S1 Overview of the distribution of starter cells in the ACC. Schematic representation of starter cells (yellow) on coronal sections from anterior to posterior ACC.

FIGURE S2 Control experiment demonstrating specificity of retrograde trans-synaptic tracing approach. **(A)** Timeline of virus injection into the ACC of C57BL/6 mice for retrograde trans-synaptic tracing. **(B)** The diagram of the ACC (injection site). Red box indicates the regions in **(C)**. **(C)** Representative confocal images of GFP^+^ neurons in C57BL/6 mice and CRH-ires-Cre mice. No evidence of TVA-GFP fluorescence positive cells was observed in C57BL/6 mice. Scale bars, 100 μm.

FIGURE S3 Distribution of input neurons in the ORB, RSP, AM and MD. The rabies virus-labeled cells are shown in red. Scale bar, 100 μm. AId, agranular insular arear, dorsal part; AON, anterior olfactory nucleus; MOB, main olfactory bulb; SUBd, subiculum, dorsal part; CM, central medial nucleus of the thalamus; IAD, interanterodorsal nucleus of the thalamus; AV, anteroventral nucleus of thalamus; PVT, paraventricular nucleus of the thalamus; MH, medial habenula; LH, lateral habenula; sm, stria medullaris; AD, anterodorsal nucleus; IMD, Intermediodorsal nucleus of the thalamus.

FIGURE S4 Layer specificity of diverse cortical inputs. Representative confocal images of input neurons in the MO, SS, PTLp, and VIS. Scale bar, 100 μm.
