# Supplementary figures and images for: Whole-Brain Mapping of Monosynaptic Afferent Inputs to Cortical CRH Neurons
Source: Front Neurosci. 2019 Jun 4;13:565. doi: 10.3389/fnins.2019.00565 (PMC6558184; doi:10.3389/fnins.2019.00565)

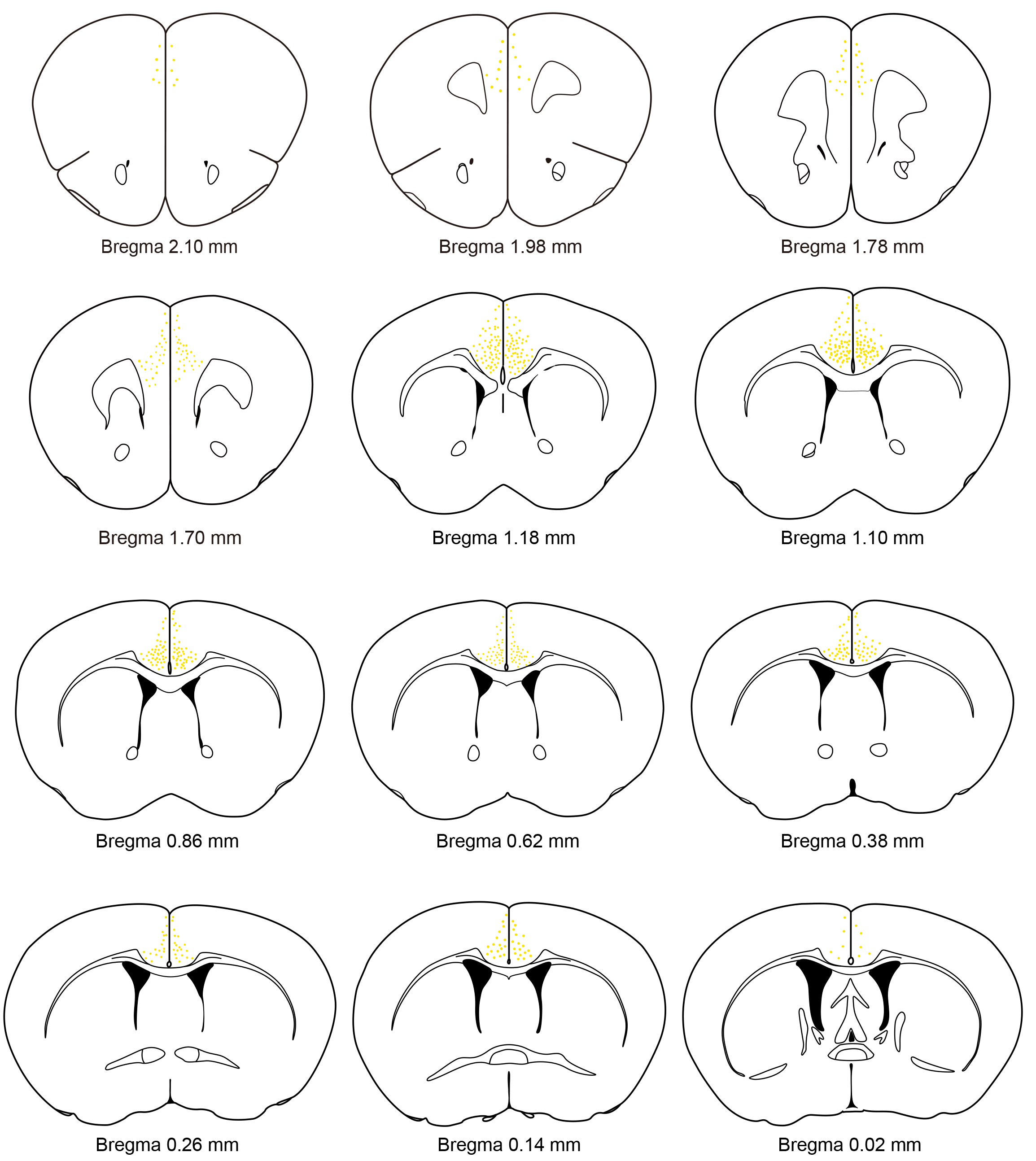

Supplement: FIGURE S1 — Overview of the distribution of starter cells in the ACC. Schematic representation of starter cells (yellow) on coronal sections from anterior to posterior ACC. [file Image_1.JPEG]

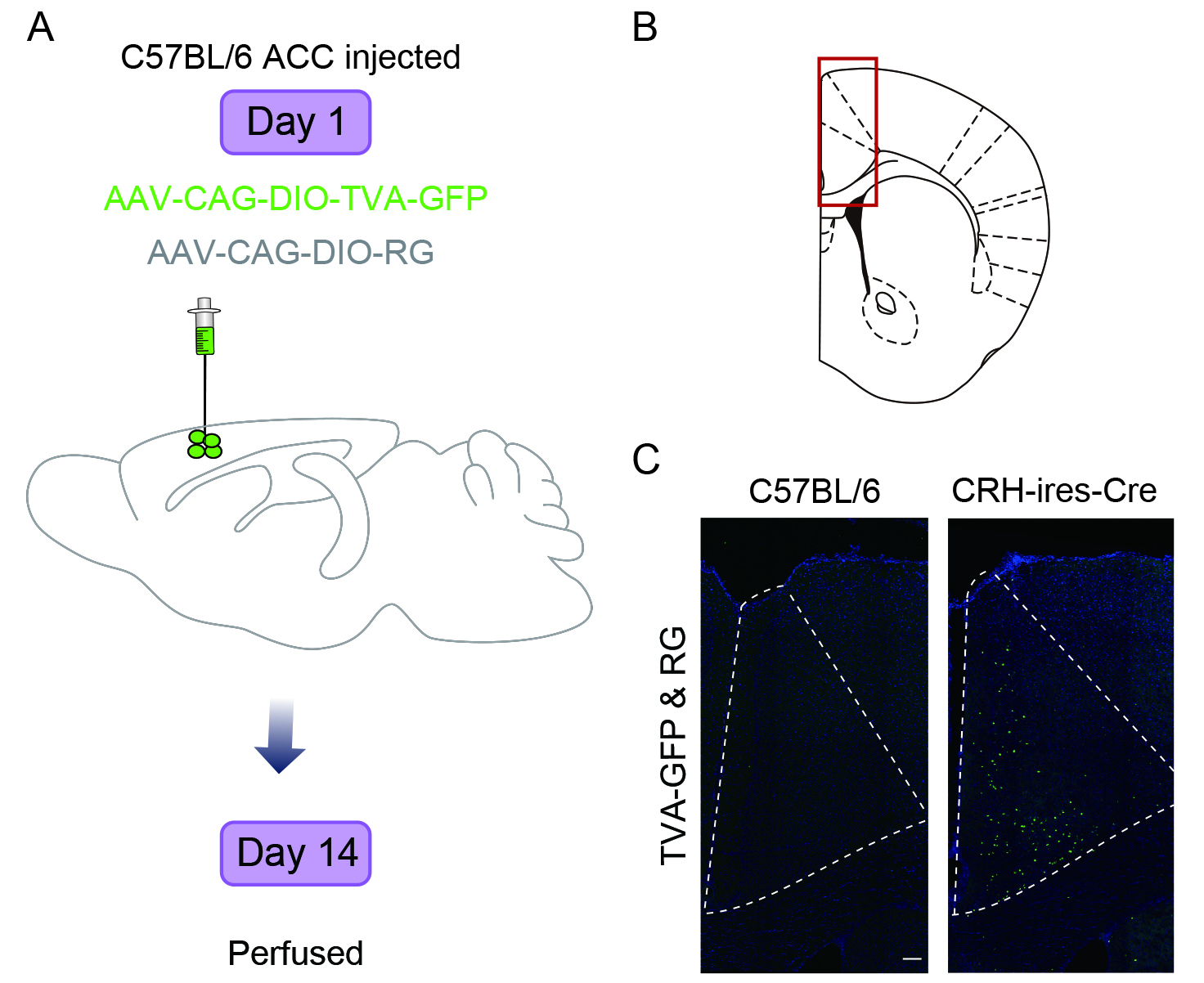

Supplement: FIGURE S2 — Control experiment demonstrating specificity of retrograde trans-synaptic tracing approach. (A) Timeline of virus injection into the ACC of C57BL/6 mice for retrograde trans-synaptic tracing. (B) The diagram of the ACC (injection site). Red box indicates the regions in (C). (C) Representative confocal images of GFP+ neurons in C57BL/6 mice and CRH-ires-Cre mice. No evidence of TVA-GFP fluorescence positive cells was observed in C57BL/6 mice. Scale bars, 100 μm. [file Image_2.JPEG]

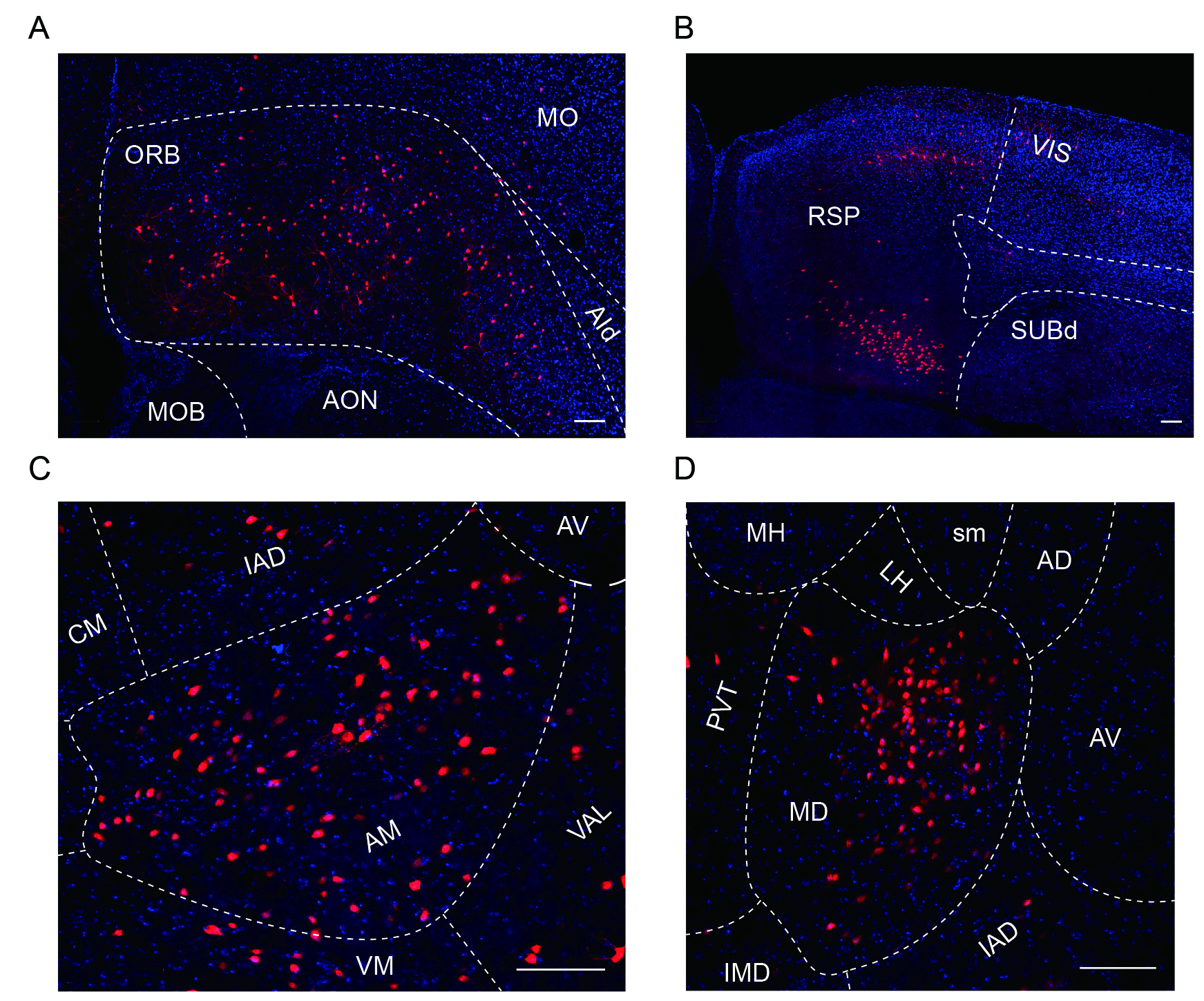

Supplement: FIGURE S3 — Distribution of input neurons in the ORB, RSP, AM and MD. The rabies virus-labeled cells are shown in red. Scale bar, 100 μm. AId, agranular insular arear, dorsal part; AON, anterior olfactory nucleus; MOB, main olfactory bulb; SUBd, subiculum, dorsal part; CM, central medial nucleus of the thalamus; IAD, interanterodorsal nucleus of the thalamus; AV, anteroventral nucleus of thalamus; PVT, paraventricular nucleus of the thalamus; MH, medial habenula; LH, lateral habenula; sm, stria medullaris; AD, anterodorsal nucleus; IMD, Intermediodorsal nucleus of the thalamus. [file Image_3.JPEG]

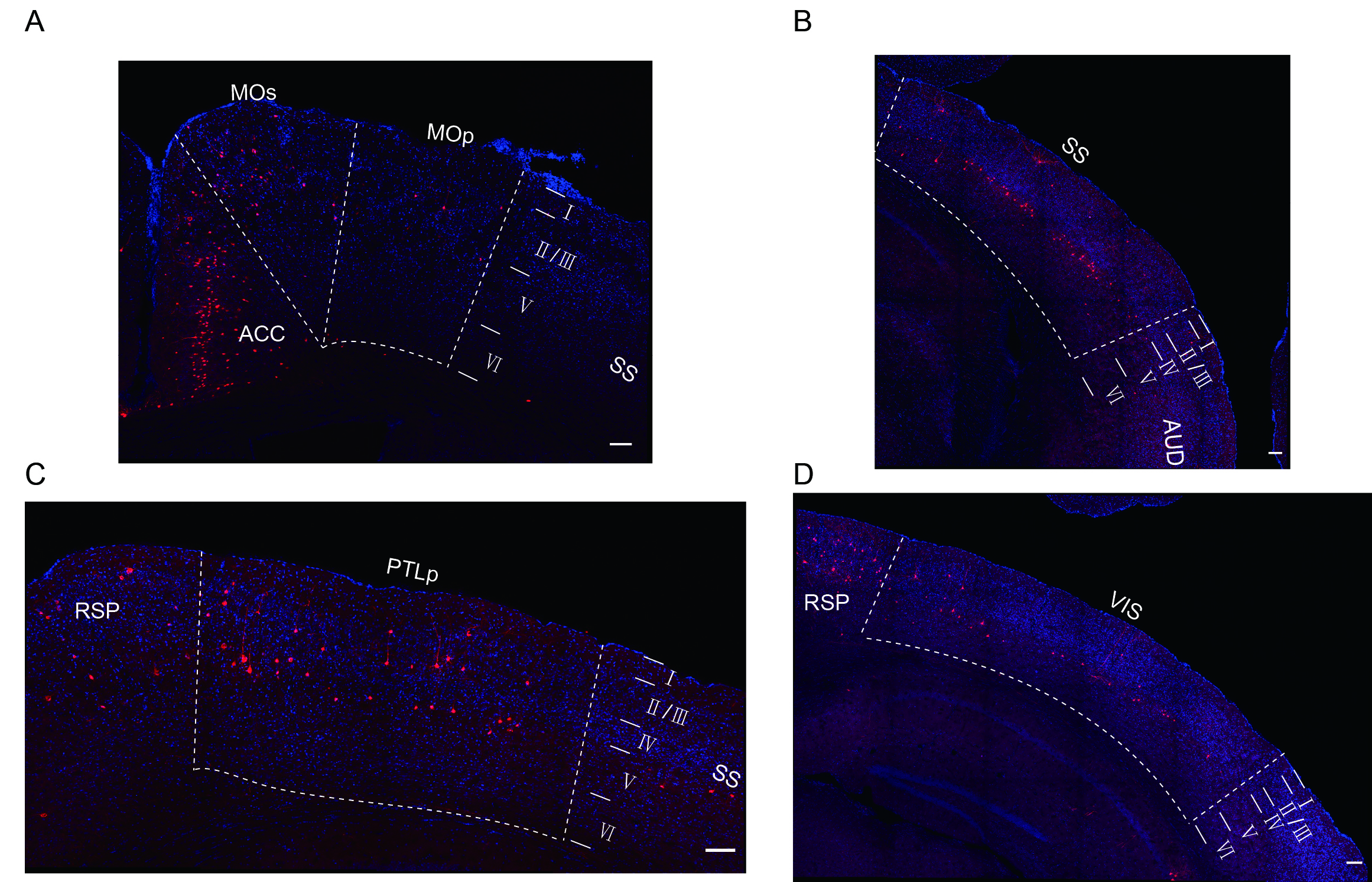

Supplement: FIGURE S4 — Layer specificity of diverse cortical inputs. Representative confocal images of input neurons in the MO, SS, PTLp, and VIS. Scale bar, 100 μm. [file Image_4.JPEG]
